# Enhanced production of antifungal lipopeptide iturin A by *Bacillus amyloliquefaciens* LL3 through metabolic engineering and culture conditions optimization

**DOI:** 10.1186/s12934-019-1121-1

**Published:** 2019-04-10

**Authors:** Yulei Dang, Fengjie Zhao, Xiangsheng Liu, Xu Fan, Rui Huang, Weixia Gao, Shufang Wang, Chao Yang

**Affiliations:** 1 Key Laboratory of Molecular Microbiology and Technology for Ministry of Education, Nankai University, Tianjin, 300071 China; 2 State Key Laboratory of Medicinal Chemical Biology, Nankai University, Tianjin, 300071 China

**Keywords:** Iturin A, *Bacillus amyloliquefaciens*, Promoter substitution, Response surface methodology, Pleiotropic regulator

## Abstract

**Background:**

Iturins, which belong to antibiotic cyclic lipopeptides mainly produced by *Bacillus* sp., have the potential for application in biomedicine and biocontrol because of their hemolytic and antifungal properties. *Bacillus amyloliquefaciens* LL3, isolated previously by our lab, possesses a complete iturin A biosynthetic pathway as shown by genomic analysis. Nevertheless, iturin A could not be synthesized by strain LL3, possibly resulting from low transcription level of the *itu* operon.

**Results:**

In this work, enhanced transcription of the iturin A biosynthetic genes was implemented by inserting a strong constitutive promoter C2up into upstream of the *itu* operon, leading to the production of iturin A with a titer of 37.35 mg l^−1^. Liquid chromatography-mass spectrometry analyses demonstrated that the strain produced four iturin A homologs with molecular ion peaks at *m*/*z* 1044, 1058, 1072 and 1086 corresponding to [C_14_ + 2H]^2+^, [C_15_ + 2H]^2+^, [C_16_ + 2H]^2+^ and [C_17_ + 2H]^2+^. The iturin A extract exhibited strong inhibitory activity against several common plant pathogens. The yield of iturin A was improved to 99.73 mg l^−1^ by the optimization of the fermentation conditions using a response surface methodology. Furthermore, the yield of iturin A was increased to 113.1 mg l^−1^ by overexpression of a pleiotropic regulator DegQ.

**Conclusions:**

To our knowledge, this is the first report on simultaneous production of four iturin A homologs (C_14_–C_17_) by a *Bacillus* strain. In addition, this study suggests that metabolic engineering in combination with culture conditions optimization may be a feasible method for enhanced production of bacterial secondary metabolites.

**Electronic supplementary material:**

The online version of this article (10.1186/s12934-019-1121-1) contains supplementary material, which is available to authorized users.

## Background

Iturin A, which contains a β-amino fatty acid chain with 14–17 carbons and a cyclic heptapeptide, is a potent antifungal lipopeptide mainly produced by *Bacillus* sp. [1–3]. Iturin A can be used as a potential biopesticide against plant pathogens and used for the treatment of human and animal mycoses due to its low toxicity and the lack of allergic effects on the host [4–6].

Iturin A is biosynthesized by the non-ribosomal peptide synthetases (NRPSs), and the Iturin A biosynthetic operon consists of four open reading frames called *ituD*, *ituA*, *ituB* and *ituC* [7]. *ituD* encodes a malonyl-CoA transacylase (MCT-domain), which is related to the synthesis of fatty acids. *ituA*, *ituB* and *ituC* contain amino acid-activating modules that encode the peptide and subunits of bacillomycin D synthetase. In addition, a TE domain is located at the C-terminal end of *ituC*, which is responsible for the cyclization and release of the lipoheptapeptide intermediate [3, 8]. So far, several iturin A-producing strains have been reported, including *Bacillus subtilis*, *Bacillus amyloliquefaciens*, *Bacillus licheniformis*, *Bacillus thuringiensis* and *Bacillus methyltrophicus* [7, 9–16].

Currently, promoter engineering can serve as a powerful tool for fine-tuning of the activity of the biosynthetic pathway enzymes for enhanced synthesis of many valuable products. In a previous study, the yield of iturin A was increased by threefold in *B. subtilis* RB14 by the replacement of the native promoter of the iturin A operon by a promoter of the plasmid pUB110 replication protein P_*repU*_ [7]. In another study, through the substitution of the surfactin operon promoter for a native lichenysin biosynthesis operon promoter, the mutant *B. licheniformis* WX02-Psrflch produced 779 mg l^−1^ lichenysin, a 5.5-fold improvement compared to that of wild strain WX-02 (121 mg l^−1^), indicating that enhanced production of lichenysin in WX02-Psrflch was attributed to elevated expression of lichenysin biosynthetic genes [17]. Recently, Jiao et al. [18] constructed an artificial IPTG-inducible promoter Pg2 and further introduced two point mutations in the − 35 and − 10 regions to produce a strong promoter Pg3. Pg3 was substituted for the native surfactin synthase promoter in the genome of *B. subtilis* THY-7, resulting in an engineered strain with a surfactin titer of 9.74 g l^−1^, a 16.7-fold improvement compared to that of wild-type strain THY-7.

Optimization of fermentation process is considered as a useful strategy to improve the yield of iturin A. In *Bacillus* sp. BH072, the yield of iturin A was improved to 52.21 mg ml^−1^ by optimizing medium components and fermentation conditions using a one-factor test and response surface methodology (RSM) [19]. In *B. subtilis* 3–10, significant enhancement of iturin A production could be achieved by a novel two-stage stepwise decreased glucose feeding strategy, leading to a maximum yield of 1.12 g l^−1^ [20].

Antibiotic lipopeptide biosynthesis in *Bacillus* strains is under regulation of complex metabolic network [8]. The biosynthesis of the antibiotic substances in *Bacillus* strains is regulated by the specific pleiotropic regulators [13, 21, 22]. For example, the quorum sensing cluster *comQXPA* plays important roles in surfactin biosynthesis. ComP activated by ComX phosphorylates the response regulator ComA, and then ComA motivates transcription of the *srfA* operon through binding to the promoter P_*srf*_ [23]. It was demonstrated that both the three pleiotropic regulators (DegU, DegQ and ComA) and two sigma factors (σB and σH) positively regulate transcriptional activation of the *bmy* promoter to promote bacillomycin D synthesis [21]. It was also verified that membrane spanning protein YczE and 4′-phosphopantetheinyl transferase Sfp exert their effect on bacillomycin D synthesis in a posttranscriptional manner [24]. A recent study indicated that the expression level of AbrB and PhrC was reduced with increased bacillomycin D production [25].

*Bacillus amyloliquefaciens* LL3, which was identified initially as a poly-γ-glutamicacid (γ-PGA)-producing strain, has been shown to possess the complete Iturin A biosynthetic pathway in the genome based on the genome data of strain LL3 [26, 27], suggesting that this strain has the potential to produce iturin A. In this work, the production of antifungal lipopeptide iturin A in *B. amyloliquefaciens* LL3 was achieved by enhanced expression of the iturin A biosynthetic pathway. Furthermore, fermentation conditions optimization was carried out by using an RSM to improve the yield of iturin A. Finally, the production of iturin A was further enhanced by overexpression of a pleiotropic regulator DegQ.

## Results

### Genome and transcriptome analysis of *B. amyloliquefaciens*

**Fig. 1:**
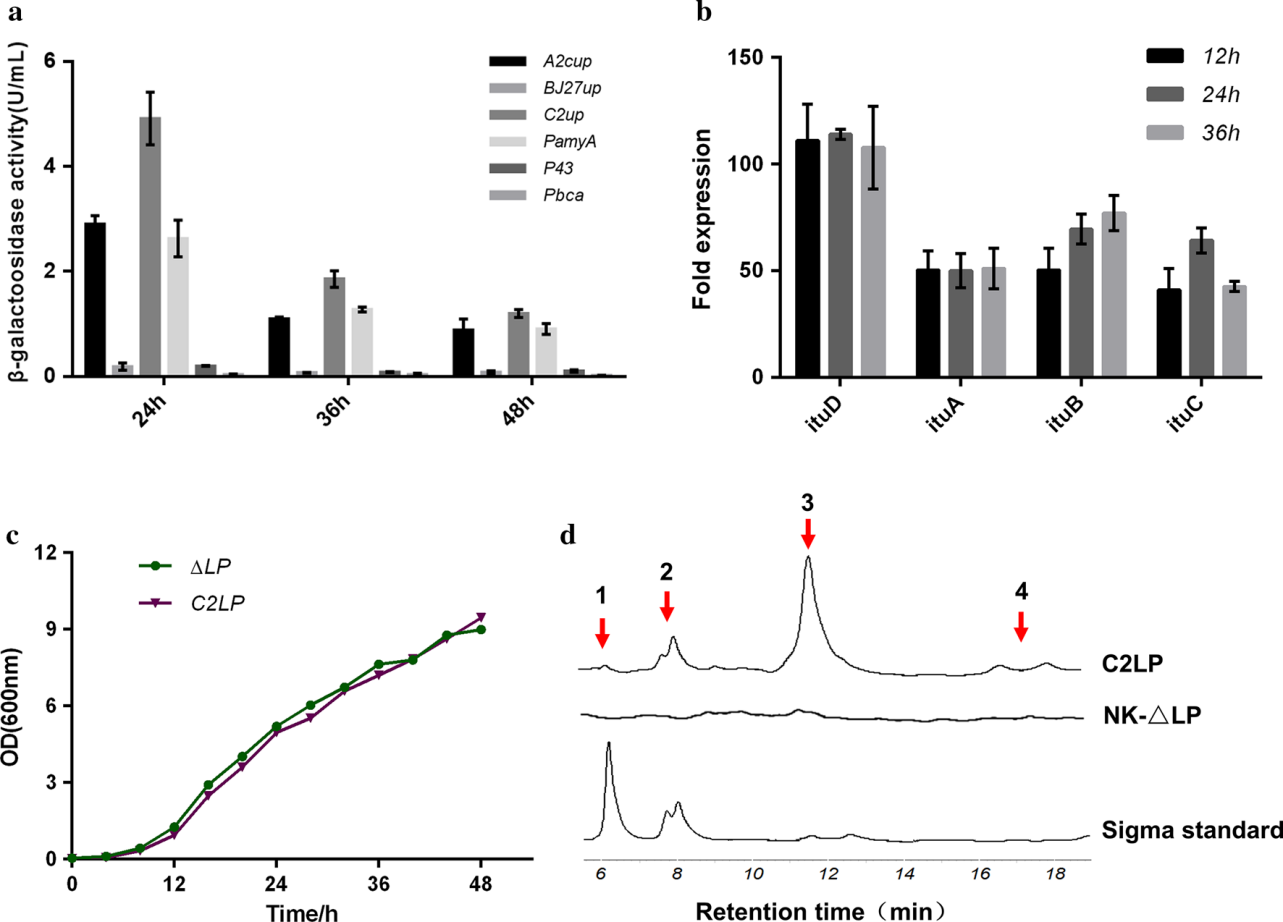
Strength comparison of the selected promoters and the effects of promoter C2up on the *itu* operon transcription and iturin A production. **a** Quantification of P_A2cup_-, P_BJ27up_-, P_C2up_-, P_*amyA*_- P_43_- and P_*bca*_-driven β-galactosidase (*bgaB*) activity at 24, 36 and 48 h; **b** changes in the transcriptional level of *ituD*, *ituA*, *ituB* and *ituC* after 12, 24 and 36 h of fermentation, and the transcriptional level of each *itu* gene in NK-∆LP was used as a control; **c** growth curves of *B. amyloliquefaciens* NK-∆LP and C2LP; **d** HPLC chromatograms of Sigma standard and the produced iturin A samples by C2LP, and NK-∆LP was used as a control. Data are mean values ± standard deviations of three replicates

According to the genome information, the six antibiotic substance biosynthetic gene clusters with a total size of ~ 170 kb, i.e., bacillaene, bacillibactin, bacilysin, iturin A, surfactin and fengycin, are present in the genome of *B. amyloliquefaciens* NK-1, accounting for 4.26% of its total genetic capacity (Additional file 1: Figure S1). Among the gene clusters, the *itu* biosynthetic gene cluster (38 kb) including *ituD*, *ituA*, *ituB* and *ituC* is responsible for iturin A biosynthesis and shows high homology to that of *B. amyloliquefacians* DSM7. A mutant strain NK-∆LP was generated from the strain NK-1 by deleting the γ-PGA synthetase gene cluster. Transcriptome analysis indicated that the transcriptional levels of the *ituD*, *ituA*, *ituB* and *ituC* in NK-∆LP were 1.9-, 4.59-, 4.39- and 4.22-fold higher than that in NK-1, respectively (Table 1). The fragments per kilobase million (FPKM) can be used as an indicator of the transcriptional activity of a gene [28]. Transcriptional activity of a gene is positively correlated with FPKM value. For NK-∆LP, the FPKM values for the *ituD*, *ituA*, *ituB* and *ituC* were still very low (Table 1). The low transcriptional level may contribute to the fact that iturin A was not detected in the culture broth of NK-∆LP when using the original or modified Landy medium (Fig. 1).

**Table 1:** Expression level comparison of genes in NK-1 and ∆LP

Gene	Protein	NK-1 FPKM	∆LP FPKM	Expression level fold change
*ituD*	Malonyl-CoA transacylase	6.07445	11.3028	1.90
*ituA*	Iturin A synthetase A	4.33389	52.1055	4.59
*ituB*	Iturin A synthetase B	2.82559	29.7206	4.39
*ituC*	Iturin A synthetase C	6.89153	64.1951	4.22
*ywsC*	PGA synthase CapB	37.3213	–	–
*amyA*	Alpha-amylase	374.999	2554.04	3.77

*FPKM* fragments per kilobase of transcript per million fragments mapped

### Selection of strong promoter to construct an iturin A-producing strain *B. amyloliquefaciens* C2LP

The strengths of the six promoters selected in this study were characterized by a β-galactosidase reporter assay. The β-galactosidase activity revealed that the transcriptional activities of the six promoters showed considerable differences with each other, but the activity of all promoters decreased gradually over the 48-h fermentation period (Fig. 1a). By comparing the six promoters, the maximum β-galactosidase activity was observed as early as 24 h when driven by the promoter C2up, and remained high at 36 and 48 h. Consequently, we further tested the ability of the screened strongest promoter C2up to improve the production of iturin A.

Inserting the strong promoter C2up into upstream of the *itu* operon in the genome of NK-∆LP was achieved using a scarless genome editing method with *upp* as a counter-selectable marker [29]. Successful construction of the promoter insertion mutant *B. amyloliquefaciens* C2LP was confirmed by PCR detection using the primers C2itu-SS and C2itu-XX (Additional file 1: Figure S2). The transcriptional level of the *itu* operon in C2LP was compared with that in NK-∆LP. As expected, the transcriptional levels of all the tested *itu* biosynthetic genes in C2LP were up-regulated obviously at different growth phases (Fig. 1b). The *ituD* in C2LP showed more than 100-fold higher transcriptional level compared to the *ituD* in NK-∆LP, and the transcriptional levels of the *ituA*, *ituB* and *ituC* in C2LP were 40- to 70-fold higher than that in NK-∆LP (Fig. 1b). Both the strain C2LP and NK-∆LP showed the similar growth profiles over a 48-h incubation period and reached a maximum OD_600_ of 9.0 and 9.5 at 48 h, respectively (Fig. 1c). Meanwhile, iturin A was also detected in the fermentation broth of C2LP after 48 h of incubation (Fig. 1d). These results suggest that the strong constitutive promoter C2up may be used to enhance the transcription of the *itu* operon for iturin A production.

### Characterization of iturin A produced by *B. amyloliquefaciens* C2LP

**Fig. 2:**
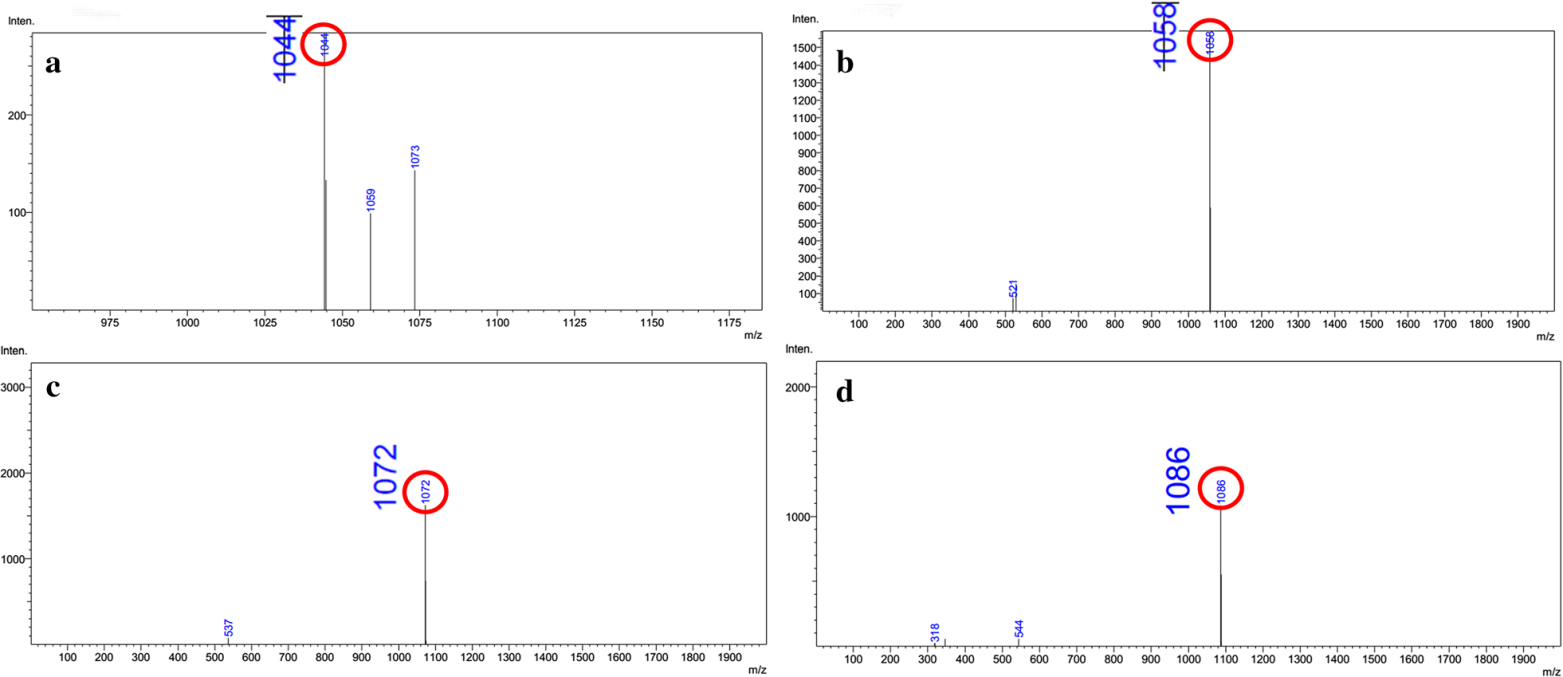
Mass spectra of the purified four iturin A homologs produced by *B. amyloliquefaciens* C2LP. **a** The peak 1 product showing a molecular ion peak at *m*/*z* 1044 corresponds to the protonated form of C_14_-β-amino acid [C_14_ + 2H]^2+^; **b** the peak 2 product showing a molecular ion peak at *m*/*z* 1058 corresponds to the protonated form of C_15_-β-amino acid [C_15_ + 2H]^2+^; **c** the peak 3 product showing a molecular ion peak at *m*/*z* 1072 corresponds to the protonated form of C_16_-β-amino acid [C_16_ + 2H]^2+^; **d** the peak 4 product showing a molecular ion peak at *m*/*z* 1086 corresponds to the protonated form of C_17_-β-amino acid [C_17_ + 2H]^2+^

It was reported that iturin A produced by bacteria is a mixture of several iturin A homologs [13]. In this study, by comparing the HPLC chromatogram of the produced iturin A by C2LP with that of the Sigma iturin A standard, the peaks 1, 2 and 3 with a retention time of about 6, 8 and 10 min, respectively, could be detected with the above two kinds of samples. In contrast, the peak 4 could be detected only with the produced iturin A by C2LP but not with the Sigma iturin A standard (Fig. 1d). To verify whether the peaks are iturin A homologs, each peak product was purified from 2000 ml of the culture supernatant of strain C2LP for LC–MS analysis. The mass spectra of the purified products had molecular ion peaks at *m*/*z* 1044, 1058, 1072 and 1086, which were attributed to [C_14_ + 2H]^2+^, [C_15_ + 2H]^2+^, [C_16_ + 2H]^2+^ and [C_17_ + 2H]^2+^, respectively (Fig. 2). These compounds are iturin A homologs which differ in their alkane chain length by 14 Da corresponding to a CH_2_ group. The peak 3 product corresponding to C_16_-β-amino acid had the highest content among the produced four iturin A homologs by C2LP (Fig. 1d).

### Antimycotic activity of iturin A produced by *B. amyloliquefaciens* C2LP

**Fig. 3:**
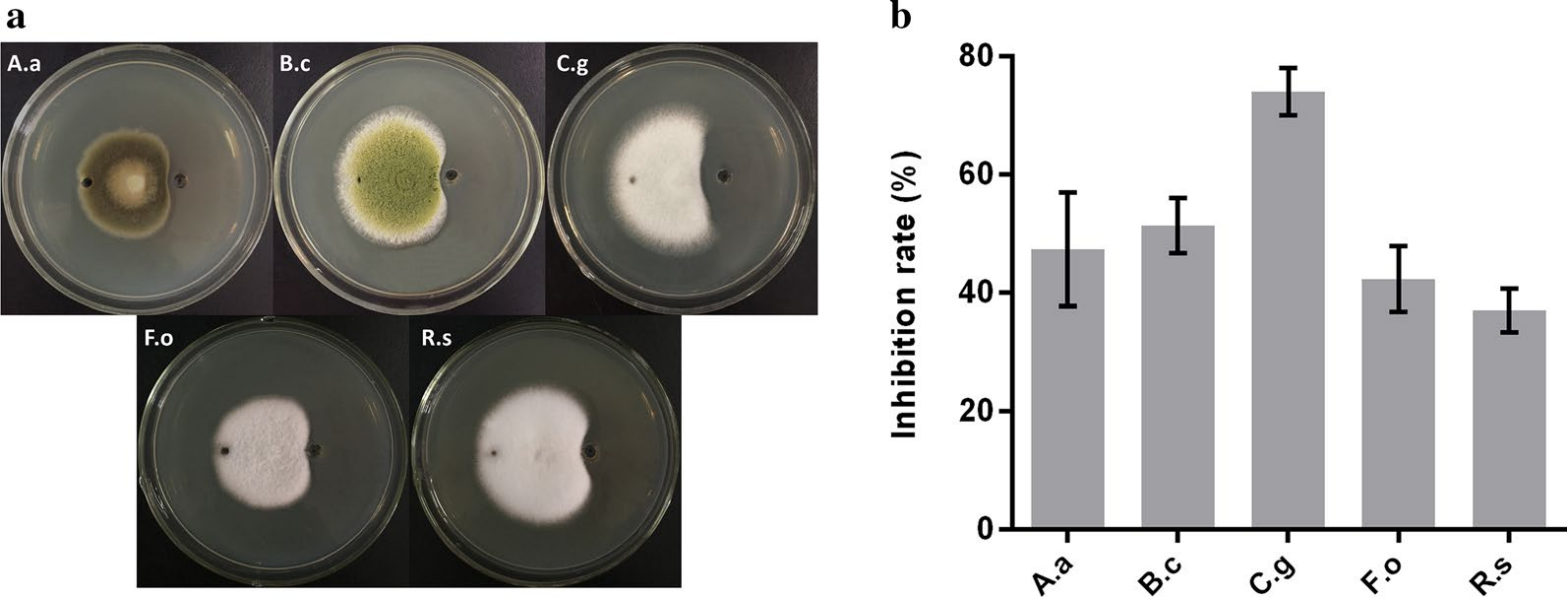
Inhibitory effect picture (**a**) and inhibition ratio (**b**) of iturin A produced by *B. amyloliquefaciens* C2LP. Samples A.a, B.c, C.g, F.o and R.s correspond to *A. alternate*, *B. cinerea*, *C. gloeosporioides*, *F. oxysporum* and *R. solani*, respectively. Each well on the right was filled with 60 μl of the iturin A extract from C2LP, and 60 μl of sterile fermentation medium as a control was added to the well on the left. Data are reported as mean values of the inhibition ratio from three independent experiments

Five fungi including *A. alternate*, *B. cinerea*, *C. gloeosporioides*, *F. oxysporum* and *R. solani* were selected to test the antifungal activity of iturin A produced by *B. amyloliquefaciens* C2LP in vitro. As a result, the five fungi were significantly inhibited by the iturin A extract after 4 days of stationary culture (Fig. 3a). The iturin A extract from *B. amyloliquefaciens* C2LP showed the strongest inhibitory activity against *C. gloeosporioides* with an inhibition ratio of 74% (Fig. 3b). In contrast, the iturin A standard (Sigma) lacking C_17_-sidechain iturin A showed a decreased inhibition ratio against *B. cinerea* and *C. gloeosporioides*.

### Pre-optimization of iturin A production by *B. amyloliquefaciens* C2LP

**Fig. 4:**
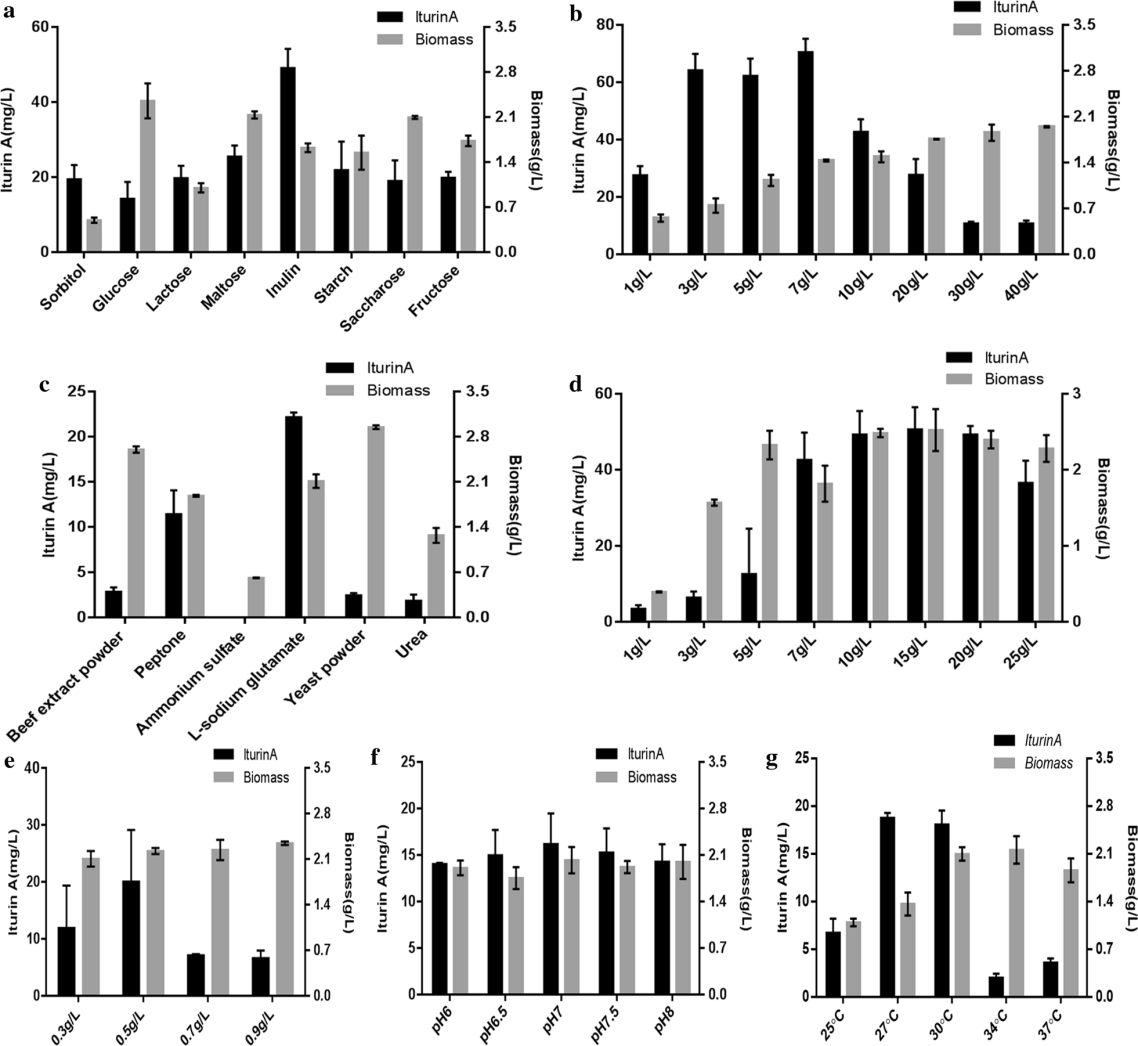
The effect of the different single factor changes on iturin A production and biomass of *B. amyloliquefaciens* C2LP. **a** Carbon source, **b** inulin concentration, **c** nitrogen source, **d** l-sodium glutamate concentration, **e** MgSO_4_ concentration, **f** initial pH of fermentation medium, **g** fermentation temperature. Data are mean values ± standard deviations of three replicates

*B. amyloliquefaciens* C2LP had an iturin A titer of 37.35 mg l^−1^ when cultured in the original Landy medium. Thus, the fermentation medium was optimized to further improve the iturin A production. The effects of carbon and nitrogen sources, pH and temperature on iturin A production were investigated in shake-flask cultures. As shown in Fig. 4, inulin was the most appropriate one among the eight carbon sources (sorbitol, glucose, lactose, maltose, inulin, starch, saccharose and fructose), and l-sodium glutamate was the best nitrogen source among the six candidates (beef extract powder, peptone, ammonium sulfate, l-sodium glutamate, yeast power and urea). The optimal pH and temperature for iturin A production by strain C2LP were 7.0 and 27 °C, respectively. We further determined the optimal concentration of the main factors. The optimal concentrations of inulin, l-sodium glutamate and MgSO_4_ were 7, 15 and 0.5 g l^−1^, respectively.

### Multiple responses optimization of iturin A production

**Fig. 5:**
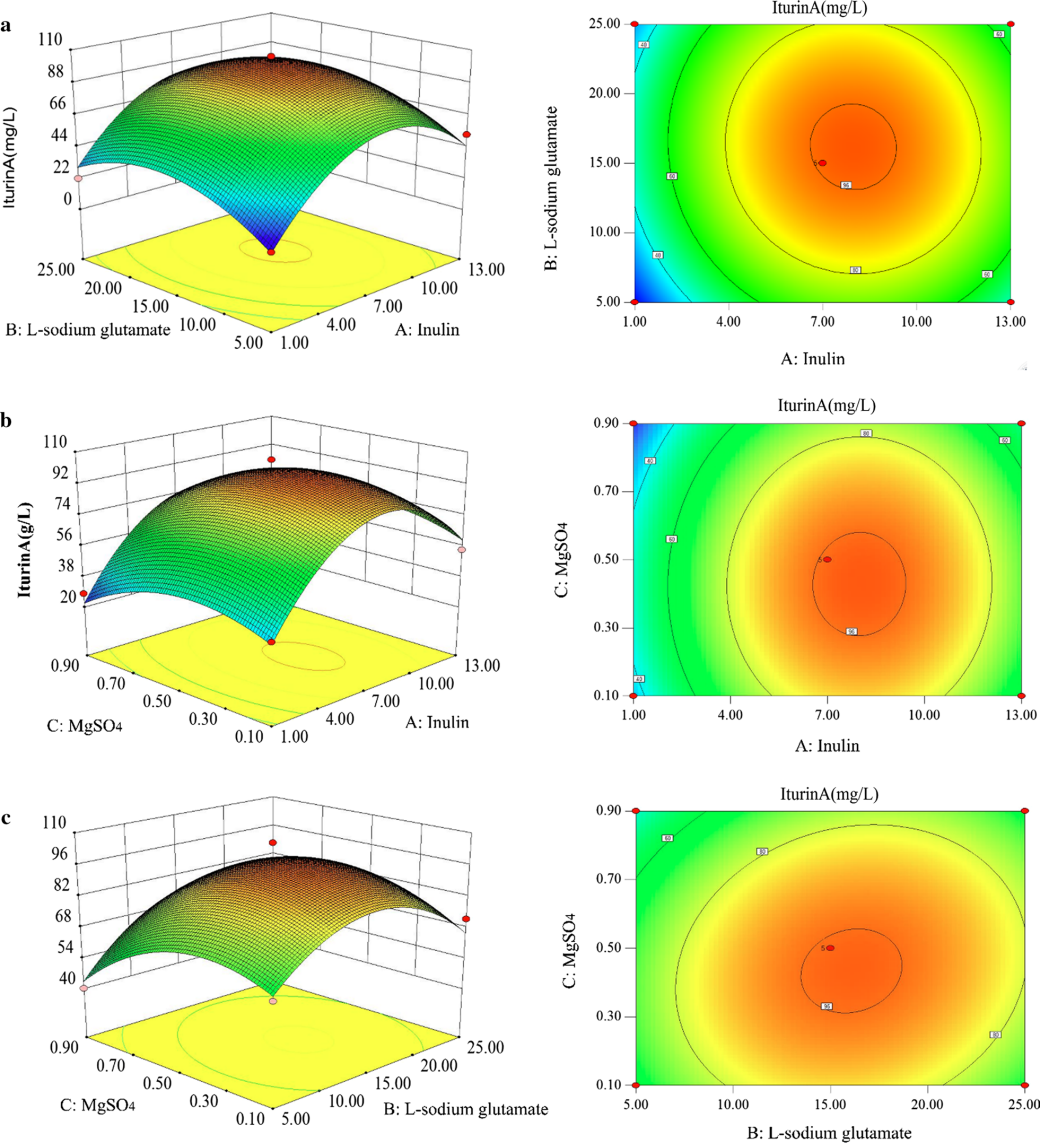
Response surface indicates the effect of three main factors on iturin A production. **a** The effect of inulin and l-sodium glutamate on iturin A production; **b** the effect of inulin and MgSO_4_ on iturin A production; **c** the effect of l-sodium glutamate and MgSO_4_ on iturin A production

In this work, to improve iturin A production, three significant influence factors including inulin, l-sodium glutamate and MgSO_4_ were selected for further optimization using the RSM design based on the single-factor tests result. The experimental design and results are shown in Table 2. Our results showed the response surfaces of two variables at the center level of other variables, respectively (Fig. 5). The non-linear nature of all response surfaces demonstrated that there were considerable interactions between each of the independent variables, and that the independent variables affected the iturin A production. From these results, we obtained the optimal concentrations of the three factors (g l^−1^): inulin 7.97, l-sodium glutamate 16.02 and MgSO_4_ 0.44. Under the optimized culture conditions, the shake-flask cultures of strain C2LP could produce 99.73 mg l^−1^ iturin A.

**Table 2:** Experiment design and results of the Box–Behnken central composite design

Run order	Factors (g/l)			Titer (mg/l)	
	A	B	C	Experimental	Predicted
1	1	5	0.5	15.70 ± 4.21	15.11
2	13	5	0.5	52.87 ± 5.07	44.98
3	1	25	0.5	22.00 ± 3.14	29.88
4	13	25	0.5	51.58 ± 1.73	52.17
5	1	15	0.1	36.43 ± 0.28	34.91
6	13	15	0.1	54.03 ± 0.51	59.80
7	1	15	0.9	28.07 ± 2.02	22.30
8	13	15	0.9	48.05 ± 0.81	49.57
9	7	5	0.1	62.02 ± 3.64	64.14
10	7	25	0.1	72.06 ± 3.71	65.69
11	7	5	0.9	36.93 ± 6.77	43.29
12	7	25	0.9	65.81 ± 7.67	63.70
13	7	15	0.5	92.76	96.83
14	7	15	0.5	96.60	96.83
15	7	15	0.5	94.20	96.83
16	7	15	0.5	105.69	96.83
17	7	15	0.5	94.89	96.83

### Effect of the overexpression and suppression of the regulatory factors on iturin A production

**Fig. 6:**
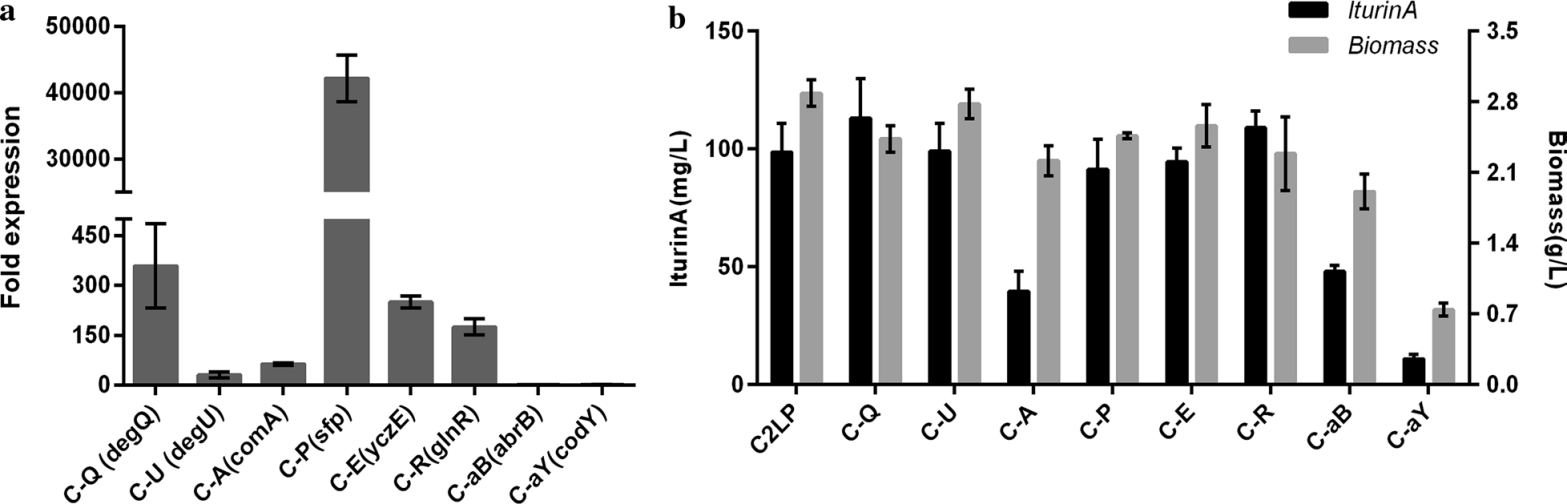
**a** Expression profiles of the eight pleiotropic regulators *degQ*, *degU*, *comA*, *sfp*, *yczE*, *glnR*, *abrB* and *codY* in the corresponding engineered strains. The expression level of each regulator in C2LP was used as a control. **b** Comparison of iturin A production and biomass of *B. amyloliquefaciens* C2LP with that of its derived strains

In this work, to test whether the overexpression or suppression of the eight regulatory factors (DegQ, DegU, ComA, Sfp, YczE, GlnR, AbrB and CodY) could directly increase the production of iturin A by strain C2LP, we constructed the various overexpression or suppression vectors and transformed the vectors into strain C2LP. All the recombinant strains, especially for C-A, C-P and C-aB, showed poor growth in the optimized Landy medium compared with strain C2LP (data not shown). As expected, the relative transcriptional levels of the eight regulatory factors were up- or down-regulated by overexpression or suppression (Fig. 6a). Moreover, the recombinant strains C-Q and C-R could produce 113.1 and 109.06 mg l^−1^ iturin A after 48 h of cultivation, respectively, which were 14.51 and 10.46% higher than the iturin A titer of strain C2LP. In contrast, the recombinant strains C-A, C-aB and C-aY showed a sharp decrease in the iturin A titer, only with a titer of 39.54, 48.12 and 10.95 mg l^−1^, respectively (Fig. 6b). Generally, the functionality of pleiotropic regulators would be affected by culture conditions. Nevertheless, the iturin A titer was not further improved by applying the RSM method to the finally obtained degQ-overexpressing strain C-Q.

## Discussion

*B. amyloliquefaciens* LL3 is a natural γ-PGA producing strain. In our previous study, we could not detect any iturin A product in the culture broth of the wild strain *B. amyloliquefaciens* LL3 even though this strain contains the complete *itu* operon [26]. Since both the γ-PGA and iturin A biosynthetic pathways may compete for the same substrate glutamic acid, we constructed the γ-PGA sythetase knockout strain NK-∆LP [26]. Unfortunately, no iturin A could be detected in the culture broth of strain NK-∆LP (Fig. 1d).

Due to the key roles of the promoter in the regulation of gene expression, many studies focused on promoter modification and substitution [17, 18, 30]. In strain NK-∆LP, the low transcriptional level of the *itu* operon, as revealed by transcriptome analysis (Table 1), might be the main reason of the block of iturin A production. Therefore, the replacement of the native iturin synthetase promoter with a strong *Bacillus* constitutive promoter may be a feasible approach for enhancing the transcriptional level of the *itu* operon in strain NK-∆LP.

In this study, the six strong *Bacillus* constitutive promoters (A2cup, BJ27up, C2up, P_*amyA*_, P43 and P_*bca*_) were selected based on previous literatures and transcriptome analysis. P43, a strong constitutive promoter, is widely used in *Bacillus* for enzyme expression or pathway engineering, such as the overexpression of endoglucanase, α-amylase and keratinase [31–33], as well as hyaluronan synthesis [34]. A2cup and C2up from *B. subtilis* phage φ29 are strong early σA-RNA polymerase-dependent promoters [35]. BJ27up is a strong constitutive promoter from *Bacillus* expression plasmid pJH27. P_*bca*_ and P_*amyA*_ derived from *B. amyloliquefaciens* NK-1, which are responsible for the transcription of γ-PGA synthetase and α-amylase, respectively, were assumed to possess higher transcriptional activity than the native iturin synthetase promoter, as judged by FPKM values from transcriptome analysis (Table 1). C2up was demonstrated to be the strongest promoter among the above six promoters using β-galactosidase reporter assays (Fig. 1a). Integrating the C2up into upstream of the *itu* operon in the genome of NK-∆LP could significantly elevate transcriptional level of the *itu* operon at different growth phases (Fig. 1b). As expected, the C2up substitution strain C2LP was able to produce iturin A with a titer of 37.35 mg l^−1^ (Fig. 1d). Taken together, these results strongly suggest that the substitution of the strong *Bacillus* constitutive promoters C2up for the native iturin synthetase promoter may promote the production of iturin A by enhancing the transcriptional level of the *itu* operon.

Optimization of fermentation medium and condition may serve as an effective approach for the improvement of the antifungal lipopeptide production. In a previous study, carbon and nitrogen sources were demonstrated to be the main factors capable of enhancing iturin A production, and their optimum amounts in the fermentation medium were predicted [5]. Optimization of iturin A production by adding Asn, Glu and Pro during the fed-batch fermentation process was studied using an artificial neural network-genetic algorithm and uniform design. The iturin A yield was improved to 13,364.5 ± 271.3 U ml^−1^, which was 1.34-fold higher than that of the control (batch fermentation without adding the amino acids) [6]. It was reported that inulin could promote efficient production of bacillomycin D by significantly improving the expression of lipopeptide bacillomycin D synthetase genes and by up-regulating the related regulatory factors such as ComA, DegU, SigmaH and Spo0A [25]. Coincidentally, fructose similarly enhanced the expression of lipopeptide fengycin synthetase genes, regulatory factors (ComA, SigmaH, DegU and DegQ) and the cell division and growth related genes (*ftsZ* and *divIVA*), leading to increased fengycin production [36]. In this study, five single factors including both fermentation conditions and medium components were explored to select the appropriate degree, and finally it was confirmed that inulin 7 g l^−1^, l-sodium glutamate 15 g l^−1^, MgSO_4_ 0.5 g l^−1^, pH 7.0 and temperature 27 °C were the appropriate one (Fig. 4). RSM is a time-saving experimental methodology that can use quantitative data to evaluate multiple process variables and their interactions by establishing a mathematical model [37, 38]. RSM has been employed to optimize the fermentation conditions and media components for cyclic lipopeptide production in shake-flask fermentation [19, 39]. In this study, three significant influence factors inulin, l-sodium glutamate and MgSO_4_ were chosen to be optimized by RSM for rapidly achieving the optimum fermentation medium. The three factors optimized by RSM were as follows: inulin (A) 7.97 g l^−1^, l-sodium glutamate (B) 16.02 g l^−1^ and MgSO_4_ (C) 0.44 g l^−1^ (Fig. 5). The result is definitely believable because the coefficient of determination (*R*^2^) of the model was 0.9696 (very close to 1).

So far, the regulatory mechanism of iturin A biosynthesis is still unknown. In contrast, the regulatory mechanism of bacillomycin D biosynthesis has been studied preliminarily [21, 24, 25]. Since iturin A and bacillomycin D belong to iturins family and their synthetase operons exhibit high similarity, the biosynthesis of iturin A and bacillomycin D may be similarly regulated. Although *B. subtilis* 168 was converted into an iturin A producer RM/iS2 by the introduction of the iturin A operon and an *sfp* gene, the titer of iturin A was very low. By inserting the pleiotropic regulator *degQ*, the titer of iturin A was increased to 64 μg ml^−1^, which was eightfold higher than that of RM/iS2 [13]. The *degQ* gene from *B. subtilis* 168 is not functional because of the mutation in promoter region. In this study, to explore whether overexpression or repression of pleiotropic regulators is profitable for iturin A synthesis, we selected the eight pleiotropic regulators DegQ, DegU, ComA, GlnR, CodY, AbrB, YczE and Sfp from *B. amyloliquefaciens* LL3. As shown in Fig. 6b, DegQ had the most significant positive impact on iturin A production, followed by GlnR, reaching a titer of 113.1 and 109.1 mg l^−1^, respectively. In the future, more deep studies on the regulatory mechanism of iturin A biosynthesis are required for improved production of iturin A by manipulating the specific regulatory factors.

The composition of iturins produced differs in their alkane chain length and would largely depend on the species of *Bacillus* used. The main components of iturin A produced by the *B. subtilis* 168-derived RM/iSd series strains were C_14_- and C_15_-β-amino acid and a minor amount of C_16_-β-amino acid was also detected [13]. Chen et al. isolated an active substance from *B. subtilis* JA by reversed-phase HPLC separation and identified two iturin A homologs (C_14_- and C_15_-β-amino acid) by electrospray ionization and collision-induced dissociation mass spectrometry analysis [40]. *B. methyltrophicus* TEB1 produced three iturin A homologs C_14_-, C_15_- and C_16_-β-amino acid, among which C_15_-β-amino acid was identified as the main component [10]. *B. amyloliquefaciens* W10 produced four iturin homologs, as revealed by mass spectrometry, showing molecular ion peaks at *m*/*z* 1051.5, 1065.5, 1079.5, 1081.5, 1093.5 and 1095.5, which were attributed to [C_13_ + Na]^+^, [C_14_ + Na]^+^, [C_15_ + Na]^+^, [C_14_ + K]^+^, [C_16_ + Na]^+^ and [C_15_ + K]^+^, respectively [15, 41] demonstrated the production of two iturin A homologs (*m*/*z* 1044.3, 1047.9 and 1069.5) by *B. amyloliquefaciens* B94. Caldeira et al. [42] also identified two iturin homologs (*m*/*z* 1031.5 and 1045.5) produced by *B. amyloliquefaciens* CCMI 1051. In this study, *B. amyloliquefaciens* C2LP produced four iturin A homologs C_14_-, C_15_-, C_16_- and C_17_-β-amino acid, among which C_16_-β-amino acid had the highest content followed by C_15_-β-amino acid (Figs. 1d and 2). To our knowledge, there is no report on the co-production of four iturin A homologs (C_14_–C_17_) by a *Bacillus* strain so far. The iturin A extract from *B. amyloliquefaciens* C2LP exhibited strong inhibitory activity against several common plant pathogens (Fig. 3), suggesting that the structural diversity of iturin A produced by *B. amyloliquefaciens* C2LP may contribute to a broad inhibitory spectrum against various plant pathogenic fungi.

In this study, selection of a non-natural iturin A-producing strain LL3 has three main reasons, which include: (1) strain LL3 contains all the iturin A biosynthetic genes; (2) the iturin A biosynthesis capacity can be restored by the activation of the transcription of the iturin A biosynthetic genes through the replacement of a native iturin A biosynthesis operon promoter with a strong constitutive promoter C2up; (3) *B. amyloliquefaciens* has an ability to produce diverse iturin A homologs.

## Conclusions

In this study, *B. amyloliquefaciens* LL3 was engineered to be an iturin A producer by promoter substitution. Furthermore, iturin A production was significantly improved using a combined strategy of culture conditions optimization and pleiotropic regulators overexpression. More importantly, this strain is capable of simultaneous production of four iturin A homologs, suggesting that the strain may serve as a biocontrol agent or iturin A producer for widespread application. Currently, more efforts are still needed for the elucidation of the regulatory mechnism of iturin A biosynthesis and for the industrial scale production of iturin A by the strain.

## Methods

### Strains, plasmids and culture conditions

*Escherichia coli* DH5α was used for plasmid construction and transformation. *E. coli* GM2163 was used for plasmid demethylation. The *B. amyloliquefaciens* LL3 strain was deposited in the China Center for Type Culture Collection (CCTCC) with accession number CCTCC M 208109 [27]. *B. amyloliquefaciens* NK-∆LP was used as the parental strain [43]. *B. amyloliquefaciens* C2LP was used for the production of iturin A.

Plasmid pCB containing β-galactosidase gene was used as a reporter vector for characterizing the strengths of the selected promoters [44]. A temperature-sensitive plasmid p-KSU containing a *upp* expression cassette, which was derived from the pKSV7 plasmid [45], was used for the construction of the C2up promoter insertion vector. Plasmid pHT01 was used for the overexpression and repression of pleiotropic regulators. All strains and plasmids used in this study are listed in Table 3.

**Table 3:** Strains and plasmids used in this study

Strains and plasmids	Description	Reference or source
Bacteria		
*B. amyloliquefaciens* NK-1	LL3 derivative, ∆pMC1, ∆*upp*	[43]
*B. amyloliquefaciens* NK-∆LP	NK-1 derivative, ∆*pgsBCA*	[43]
* B. subtilis*168	trpC2, containing a strong constitutive promoter P_43_	This lab
*B. amyloliquefaciens* NK-P_*bca*_	NK-1 derivative with plasmid pCB-P_*bca*_	This work
*B. amyloliquefaciens* NK-A2cup	NK-1 derivative with plasmid pCB-A2cup	This work
*B. amyloliquefaciens* NK-BJ27up	NK-1 derivative with plasmid pCB-BJ27up	This work
*B. amyloliquefaciens* NK-C2up	NK-1 derivative with plasmid pCB-C2up	This work
*B. amyloliquefaciens* NK-P_*amyA*_	NK-1 derivative with plasmid pCB-P_*amyA*_	This work
*B. amyloliquefaciens* NK-P_43_	NK-1 derivative with plasmid pCB-P_43_	This work
*B. amyloliquefaciens* C2LP	∆LP derivative, C2up promoter inserted into upstream of the *itu* operon	This work
*B. amyloliquefaciens* C-Q	C2LP derivative with plasmid pHT-*degQ*	This work
*B. amyloliquefaciens* C-U	C2LP derivative with plasmid pHT-*degU*	This work
*B. amyloliquefaciens* C-A	C2LP derivative with plasmid pHT-*comA*	This work
*B. amyloliquefaciens* C-P	C2LP derivative with plasmid pHT-*sfp*	This work
* B. amyloliquefaciens* C-E	C2LP derivative with plasmid pHT-*yczE*	This work
*B. amyloliquefaciens* C-R	C2LP derivative with plasmid pHT-*glnR*	This work
*B. amyloliquefaciens* C-aB	C2LP derivative with plasmid pHT-anti*abrB*	This work
*B. amyloliquefaciens* C-aY	C2LP derivative with plasmid pHT-anti*codY*	This work
*E. coli* DH5α	F^−^, *ϕ*80d*lac*ZM1, (*lacZYA*-*argF*)U169, *deoR*, *recA*1, *endA*1, *hsdR*17 (rk^−^, mk^+^), *phoA*, *supE*44, *λ*−*thi*-1, *gyrA*96, *relA*1	This lab
*E. coli* GM2163	F^−^, *ara*-*14 leuB6 thi*-*1 fhuA31 lacY1 tsx*-*78 galK2* *galT22 supE44 hisG4 rpsL136 (Str*^*r*^*) xyl*-*5 mtl*-*1* *dam13::*Tn9 (Cam^r^) *dcm*-*6 mcrB1 hsdR2 mcrA*	This lab
Fungi		
*Alternaria alternate*	Wild-type	This lab
*Botrytis cinerea*	Wild-type	This lab
*Colletotrichum gloeosporioides*	Wild-type	This lab
*Fusarium oxysporum*	Wild-type	This lab
*Rhizoctonia solani*	Wild-type	This lab
Plasmid		
pCB	Shuttle vector containing the β-galactosidase gene *bgaB* from *B. stearothermophilus* as a reporter gene, Amp^r^, Er^r^	Xie et al. [44]
pKSU	pKSV7 derivative with *upp* gene expression cassette	This lab
pHT01	Cm^r^; IPTG inducible expression vector for *Bacillus*	MoBiTec
pCB-P_*bca*_	pCB derivative with the promoter P_*bca*_	This work
pCB-A2cup	pCB derivative with the promoter A2cup	This work
pCB-BJ27up	pCB derivative with the promoter BJ27up	This work
pCB-C2up	pCB derivative with the promoter C2up	This work
pCB-P_*amyA*_	pCB derivative with the promoter P_*amyA*_	This work
pCB-P_43_	pCB derivative with the promoter P_43_	This work
pKSU-C2*itu*	pKSU derivative, a C2up promoter insertion vector	This work
pHT-*degQ*	pHT01 derivative with the gene *degQ*	This work
pHT-*degU*	pHT01 derivative with the gene *degU*	This work
pHT-*comA*	pHT01 derivative with the gene *comA*	This work
pHT-*sfp*	pHT01 derivative with the gene *sfp*	This work
pHT-*yczE*	pHT01 derivative with the gene *yczE*	This work
pHT-*glnR*	pHT01 derivative with the gene *glnR*	This work
pHT-*antiabrB*	pHT01 derivative with the gene anti-*abrB* sRNA sequence and *hfq* gene	This work
pHT-*anticodY*	pHT01 derivative with the gene anti-*codY* sRNA sequence and *hfq* gene	This work

All strains were cultured at 37 °C in Luria–Bertani (LB) media [46]. When required, LB media were supplemented with ampicillin (Ap, 50 μg ml^−1^), chloramphenicol (Cm, 5 μg ml^−1^) and 5-fluorouracil (5-FU, 100 μg ml^−1^). For lipopeptide iturin A production, *B. amyloliquefaciens* C2LP was cultured at 27 °C and 160 rpm for 48 h in the optimized Landy medium, pH 7.0 [47], containing 7.97 g l^−1^ inulin, 16.02 g l^−1^ l-sodium glutamate, 0.44 g l^−1^ MgSO_4_, 1 g l^−1^ KH_2_PO_4_, 0.5 g l^−1^ KCl, 0.15 mg l^−1^ FeSO_4_, 5.0 mg l^−1^ MnSO_4_ and 0.16 mg l^−1^ CuSO_4_. All fungi used in antagonism tests were incubated at 28 °C for 3 to 5 days and maintained on potato dextrose agar.

### Identification of strong promoters by β-galactosidase assay

The six strong *Bacillus* constitutive promoters (A2cup, BJ27up, C2up, P_*amyA*_, P43 and P_*bca*_) were selected based on previous literatures and transcriptome analysis of *B. amyloliquefaciens* NK-1. Transcriptome analysis of *B. amyloliquefaciens* NK-1 was carried out at BGI-Shenzhen (BGI-Shenzhen, Shenzhen, China) based on standard procedure [48]. The nucleotide sequences of the six promoters are shown in Additional file 1. To test the strengths of the selected promoters, we generated the recombinant reporter plasmids by inserting each of the promoters into upstream of the *bgaB* reporter gene on the pCB plasmid (Additional file 1: Figure S3). The NK-1 strains transformed with the reporter plasmids were used for the measurement of β-galactosidase activity using a previous method described by Xie et al. [44].

### Construction of the C2up promoter insertion mutant

For targeted insertion of the C2up promoter into upstream of the *itu* operon, the upstream and downstream homologous arms were PCR amplified from genomic DNA of *B. amyloliquefaciens* NK-∆LP using primers C2UP-F/C2UP-R and C2DN-F/C2DN-R, respectively. The C2up promoter was amplified from the pUC-C2up using primers C2-F/C2-R and then fused with the upstream and downstream fragments by overlap PCR. The fused fragment was ligated into the linearized p-KSU vector using a one-step cloning kit (Vazyme, Nanjing, China) to generate the C2up promoter insertion vector pKSU-C2*itu*.

Subsequently, we used a scar-less genome editing method to construct the promoter insertion mutant [29]. In brief, the vector pKSU-C2*itu* was transformed into *B. amyloliquefaciens* NK-∆LP by electroporation. The single-crossover mutants were screened by incubated at 42 °C for 24 h on the LB agar plates with Cm and further identified by PCR using the specific primers. The selected positive clones were then incubated in LB medium supplemented with 5-FU at 42 °C for 24 h. The cell suspensions were diluted to 10^−5^ and spread on the LB agar plates with 5-FU. The double-crossover mutants showing Cm^s^ and 5-FU^r^ were further identified by PCR, and the amplified PCR products were sent to BGI for DNA sequencing to verify the inserted promoter sequence. The resulting mutant was designated as *B. amyloliquefaciens* C2LP. All primers used in this study are listed in Additional file 1: Table S1.

### Iturin A isolation and high performance liquid chromatography (HPLC) analyses

*Bacillus amyloliquefaciens* was cultured with shaking at 160 rpm in the fermentation medium for 48 h. The cell-free culture supernatant was obtained by centrifugation at 9000 rpm and 4 °C for 20 min, subsequently acidified with 6 M HCl to pH 2 and stored overnight at 4 °C. The precipitate formed was collected by centrifugation at 4 °C and 13,000 rpm for 20 min and then resuspended with 100 ml methyl alcohol for iturin A extraction; the pH of the sample was adjusted to 7.0 using 1 M NaOH [7]. After 48 h of incubation at 180 rpm and 37 °C, the samples were centrifuged to collect the supernatant containing the iturin A extract. The supernatant was filtered through a 0.22 μm membrane filter and subjected to HPLC analysis.

For further purification of iturin A, the four iturin A homologs were recovered, respectively, at different retention times when using HPLC analysis. The collected solutions were concentrated to remove the residual acetonitrile by a vacuum rotary evaporator and then lyophilized using a vacuum freeze dryer to obtain the purified iturin A.

Lipopeptide iturin A was detected and quantified by reversed-phase HPLC as follows: 20 μl of the extract described above was injected into a HPLC C18 column [Innoval ODS-2 (4.6 mm diameter by 250 mm), Agel, Tianjin, China] maintained at 37 °C. For sample separation, the mobile phase consisting of water and acetonitrile (55:45, v/v) supplemented with 0.035% formic acid was used at a flow rate of 1 ml min^−1^. Meanwhile, the absorption spectrum was set at 220 nm. The iturin A standard (Sigma) was used for all analyses.

### Liquid chromatography-mass spectrometry (LC–MS) analyses

To determine precisely the different iturin A homologs produced by *B. amyloliquefaciens*, the extracted iturin A samples were analyzed by LC–MS using Shimadzu HPLC system LC-10AVP equipped with a photo diode array (PDA) detector and a reversed-phase C-18 column, Luna^®^ (250 mm × 4.6 mm internal diameter, 5 μ particle size, Phenomenex, USA). The samples (50 μl) were injected and two different solvent systems were used for effective separation and detection of iturin A homologs. The mobile phase consisting of water (eluent A) and acetonitrile (eluent B) supplemented with 0.035% (v/v) formic acid was used at a gradient flow rate of 10 ml min^−1^. The gradient conditions were as follows: firstly, starting at 90% eluent A and 10% eluent B, eluent A was linearly decreased to 55% with the increase of eluent B to 45% within the first 2.5 min; then, eluent A was linearly decreased to 0% with the increase of eluent B to 100% in the next 2.5 min and then maintained for 3 min; finally, eluent A was linearly increased to 90% with the decrease of eluent B to 10% in the next 1 min and then maintained for 1 min. The eluants were read at 220 nm using PDA detector (UVD340U, Dionex, USA). The LC-MS analyses were performed using an LTQ-XL instrument (Thermo Scientific, Germany) equipped with Xcalibur software.

### Fungal growth inhibition assay

Antifungal activity of iturin A produced by *B. amyloliquefaciens* C2LP was evaluated by the agar-well diffusion method using the five fungi *Alternaria alternate*, *Botrytis cinerea*, *Colletotrichum gloeosporioides*, *Fusarium oxysporum* and *Rhizoctonia solani* [15]. Wells of 5-mm diameter were formed in PDA agar plates with a sterile cork borer. Then, the iturin A extract (60 μl) from *B. amyloliquefaciens* C2LP was added into the well. Sterile fermentation medium (60 μl) was added to another well, which was used as a control. A 5-mm diameter disk inoculated with fungi was placed in the center of the agar plate, and then the plates were incubated at 28 °C and monitored for any inhibition of mycelia growth during a 3- to 5-day period.

### Single-factor tests and multiple responses optimization

To obtain a higher iturin A yield, single-factor tests and RSM were used to optimize the fermentation medium. In this study, the Box-Behnken experimental design model was used. Our experimental plan consisted of 17 trials and the independent variables were studied at three different levels, designated as − 1, 0 and + 1 for low, middle and high values, respectively (Additional file 1: Table S2). All the experiments were carried out in triplicate and the average yield of iturin A obtained was taken as the dependent variable or response. The software Design-Expert 8.0.6.1 (Stat-Ease, USA) was used for the experimental design.

### Overexpression and repression of pleiotropic regulators

To investigate the influence of pleiotropic regulators on iturin A production in *B. amyloliquefaciens*, the six native regulatory genes (*degQ*, *degU*, *comA*, *glnR*, *sfp* and *yczE*) from *B. amyloliquefaciens* were overexpressed using the expression vector pHT01 in *B. amyloliquefaciens* C2LP. Additionally, the two regulatory genes *codY* and *abrB* in *B. amyloliquefaciens* were repressed by the expression of synthetic small regulatory RNAs (sRNA). The sRNAs were designed and transcribed as described previously [49]. The gene IDs of *degQ*, *degU*, *comA*, *glnR*, *sfp*, *codY*, *abrB* and *yczE* are 12202327, 12205127, 12202323, 12204645, 12202653, 12204515, 12201069 and COG2364, respectively.

### RNA extraction and real-time PCR

To further determine the transcriptional level of target genes (*ituD*, *ituA*, *ituB*, *ituC*, *degQ*, *degU*, *comA*, *glnR*, *sfp*, *codY*, *abrB* and *yczE*), bacterial cultures were harvested after 24 h incubation. Total RNAs of these bacterial cultures were isolated using the commercial RNApure Bacteria kit (DNase I) (Cwbio, Beijing, China). Then, cDNA was synthesized using the extracted RNA samples and HiScript^®^ II Reverse Transcriptase SuperMix (Vazyme, Nanjing, China) according to the manufacturer’s instructions. Real-time PCR was carried out using FastStart Universal SYBR Green Master (Roche, Basel, Switzerland) on a StepOnePlus™ real-time PCR system (Applied Biosystems, Foster City, CA, USA). PCR conditions were as follows: pre-incubation at 95 °C for 10 min, followed by 40 cycles of denaturation at 95 °C for 30 s, annealing at 55 °C for 30 s and extension at 72 °C for 20 s. The transcriptional levels of target genes were normalized against that of *rspU* [50].

## Additional file

**Additional file 1: Figure S1.** Diagram of the locations for antibiotic substance gene clusters in *B. amyloliquefacians* NK-1. **Figure S2.** Confirmation of the construction of the mutant strain *B. amyloliquefaciens* C2LP via PCR. Lane M, DNA marker Ш; lane 1, PCR product obtained by amplification with the NK-∆LP genomic DNA as the template; lane 2, PCR product obtained by amplification with the C2LP genomic DNA as the template. **Figure S3.** Map of reporter vectors containing respectively the six promoters (A2up, BJ27up, C2up, P_*amyA*_, P_43_ and P_*bca*_). *bgaB*, β-galactosidase gene; Ap^R^, ampicillin resistance gene; Er^R^, erythromycin resistance gene. **Table S1.** Primers used in this study. **Table S2.** Coded and actual levels of factors used in the experimental design.

## Abbreviations

NRPSs: non-ribosomal peptide synthetases
RSM: response surface methodology
FPKM: fragments per kilobase million
HPLC: high performance liquid chromatography
LC–MS: liquid chromatography–mass spectrometry
