# Supplementary material for: Enhanced production of antifungal lipopeptide iturin A by Bacillus amyloliquefaciens LL3 through metabolic engineering and culture conditions optimization
Source: Microb Cell Fact. 2019 Apr 10;18:68. doi: 10.1186/s12934-019-1121-1 (PMC6457013; doi:10.1186/s12934-019-1121-1)
Supplement: Supplementary file 1 — Additional file 1: Figure S1. Diagram of the locations for antibiotic substance gene clusters in B. amyloliquefacians NK-1. Figure S2. Confirmation of the construction of the mutant strain B. amyloliquefaciens C2LP via PCR. Lane M, DNA marker Ш; lane 1, PCR product obtained by amplification with the NK-∆LP genomic DNA as the template; lane 2, PCR product obtained by amplification with the C2LP genomic DNA as the template. Figure S3. Map of reporter vectors containing respectively the six promoters (A2up, BJ27up, C2up, PamyA, P43 and Pbca). bgaB, β-galactosidase gene; ApR, ampicillin resistance gene; ErR, erythromycin resistance gene. Table S1. Primers used in this study. Table S2. Coded and actual levels of factors used in the experimental design. [file 12934_2019_1121_MOESM1_ESM.docx]

**Additional file 1**

Enhanced production of antifungal lipopeptide iturin A by *Bacillus amyloliquefaciens* LL3 through metabolic engineering and culture conditions optimization

Yulei Dang,^1†^ Fengjie Zhao,^1†^ Xiangsheng Liu,^1^ Xu Fan,^1^ Rui Huang,^1^ Weixia Gao,^2*^ Shufang Wang,^2*^ Chao Yang^1*^

^1^Key Laboratory of Molecular Microbiology and Technology for Ministry of Education, Nankai University, Tianjin 300071, China

^2^State Key Laboratory of Medicinal Chemical Biology, Nankai University, Tianjin 300071, China

^†^Yulei Dang and Fengjie Zhao contributed equally to this work.

^*^Correspondence to:

Weixia Gao (Tel./Fax: +86 22 23503753; E-mail: watersave@126.com)

Shufang Wang (Tel./Fax: +86 22 23503753; E-mail: wangshufang@nankai.edu.cn)

Chao Yang (Tel./Fax: +86 22 23503866; E-mail: yangc20119@nankai.edu.cn)

E-mail addresses of all the authors:

Yulei Dang (13821615007@163.com), Fengjie Zhao (zfjmail2010@163.com), Xiangsheng Liu (lxs_tianjin@163.com), Xu Fan (812382037@qq.com), Rui Huang (993014540@qq.com), Weixia Gao (watersave@126.com), Shufang Wang (wangshufang@nankai.edu.cn), Chao Yang (yangc20119@nankai.edu.cn)

**Additional Figures**

**
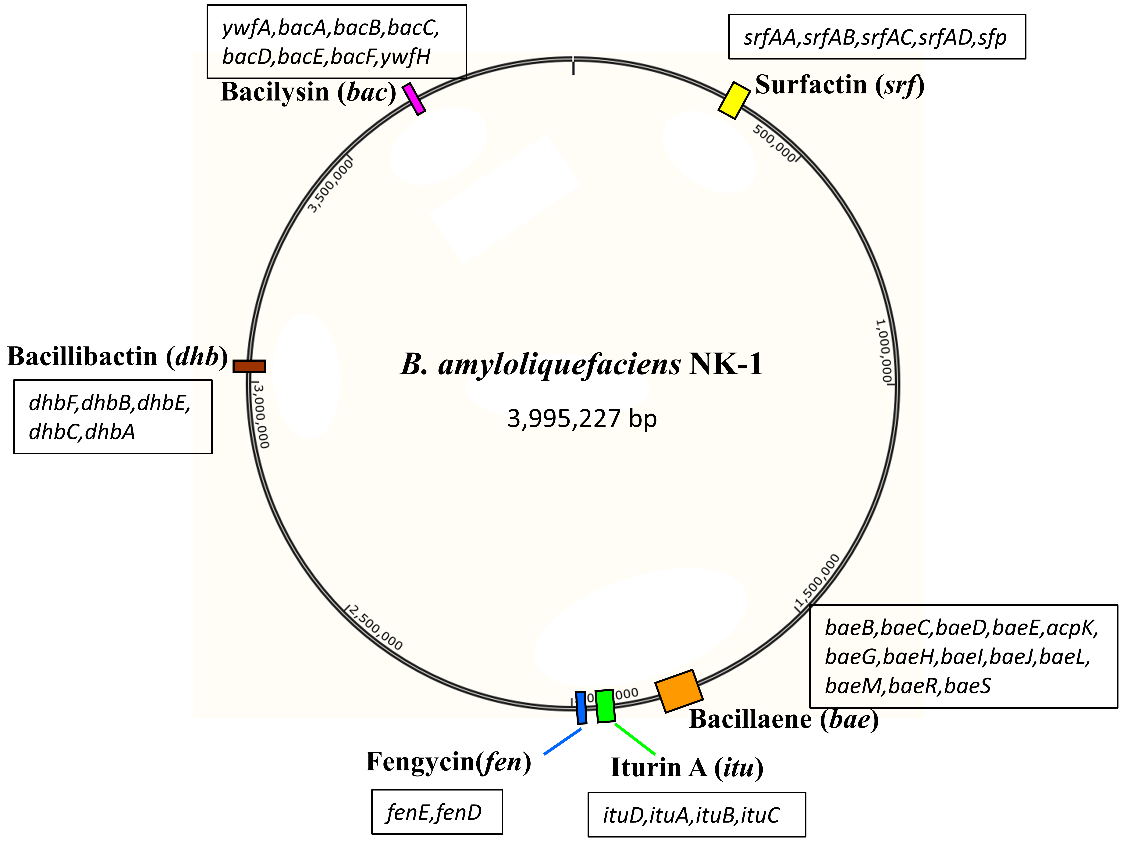
**

**Figure S1.** Diagram of the locations for antibiotic substance gene clusters in *B. amyloliquefacians* NK-1.


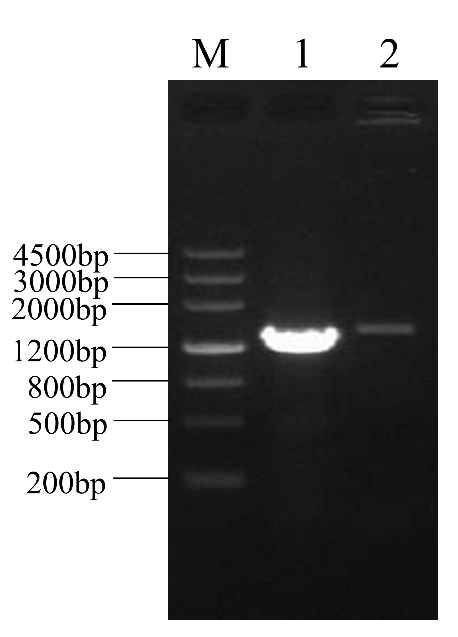


**Figure S2.** Confirmation of the construction of the mutant strain *B. amyloliquefaciens* C2LP via PCR. Lane M, DNA marker Ш; lane 1, PCR product obtained by amplification with the NK-△LP genomic DNA as the template; lane 2, PCR product obtained by amplification with the C2LP genomic DNA as the template.

**
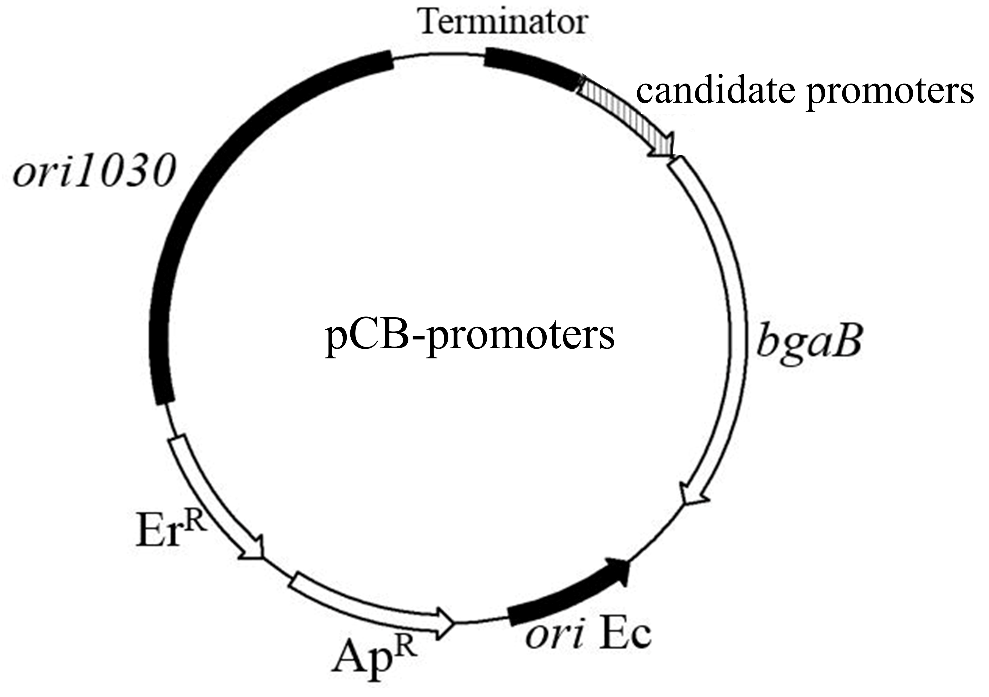
**

**Figure S3.** Map of reporter vectors containing respectively the six promoters (A2up, BJ27up, C2up, P*_amyA_*, P_43_ and P*_bca_*). *bgaB*, β-galactosidase gene; Ap^R^, ampicillin resistance gene; Er^R^, erythromycin resistance gene.

**Additional Tables**

**Table S1.** Primers used in this study

| **Primer** | **Sequence（5’→3’）** |
| --- | --- |
| P*_bca_*-F | TTAGGTACCCCAGCGGACGGAGAGTCCCG |
| P*_bca_*-R | GGTGTCGACTGATTTTTGACATCTCCTTC |
| A2cup-F | CCTGGTACCAAAATGAAATCCCCAAAAGGG |
| A2cup-R | CATGTCGACCTCTTTTATTATATCACCCTG |
| BJ27up-F | TTCCTCGAGATGGCTCCTGGTACCAAAATG |
| BJ27up-R | CATGTCGACGTGTACATTCCTCTCTTACC |
| C2up-F | GAGCTCGAGATGGCTCCTGGTACCAAAATG |
| C2up-R | CATGTCGACCTATTATAGTATAACATGTT |
| P*_amyA_*-F  P*_amyA_*-R  P_43_-F  P_43_-R  C2ituUP-F  C2ituUP-R  ituC2-F  ituC2-R  C2ituDN-F  C2ituDN-R | CCAGGTACCTCTCAGCGGAAAAAGAATCAT  GGCGTCGACTTTTCTCTCCCTCTCATTTTC  AAACTCGAGAGCTTCGTGCATGCAGGCCG  AAAGGATCCGTGTACATTCCTCTCTTACC  TAAGGATCCTCTAGAGTCGACATACAGGCTACACCTGCCGGAAAG  CATGTTATACTATAATAGGGAGGGAACTCATGAACAATCTTG  TGTTCATGAGTTCCCTCCCTATTATAGTATAACATGTTAAAC  TTGGATCTCGATGCGATCTGAGAATTCCTAACAACTAAATCAC  AGTTGTTAGGAATTCTCAGATCGCATCGAGATCCAATATCAT  TTGCATGCCTGCAGGTCGACTGAAGCAGCTCGTATTCAGCTGTG |
| C2itu-SS | GATGCCGATCGTATCATCAG |
| C2itu-XX | TGGCTGAAGAGACGAATACC |
| DegQ-F | TCTAGAGTCGACGTCCCCGGGACGTCTGCCTTTATGGTAGTTATAT |
| DegQ-R | CATTAGGCGGGCTGCCCCGGGTTAAGAAATTTTCATTGCATATGAG |
| DegU-F | TCTAGAGTCGACGTCCCCGGGTTTGTTAAATAGAGCCAAAAGAC |
| DegU-R | CATTAGGCGGGCTGCCCCGGGCTAACGCATCTCTACCCAGCCGTTT |
| ComA-F | TCTAGAGTCGACGTCCCCGGGCCAATTTATCACTAAATCGACTTGG |
| ComA-R | CATTAGGCGGGCTGCCCCGGGTTATAGTACGTTATCTGACTTGGCG |
| Sfp-F | TCTAGAGTCGACGTCCCCGGGTTTGCAGACGGAGGATCTGGACAT |
| Sfp-R | CATTAGGCGGGCTGCCCCGGGTTACAGCAGTTCTTCATACGTTTT |
| YczE-F | TCTAGAGTCGACGTCCCCGGGGAGCCGAAGAAGAAGCGAAACACTG |
| YczE-R | CATTAGGCGGGCTGCCCCGGGTTAATGAACGGACGCTGCCCGC |
| GlnR-F  GlnR-R  P-F  PabrB-R  HabrB-F  H-R  PcodY-R  HcodY-F | TCTAGAGTCGACGTCCCCGGGTTCGTTTGCAATTTTCAAAGAATTAG  CATTAGGCGGGCTGCCCCGGGTTAATGAAAGAATCGGGACATGTCA  TCTAGAGTCGACGTCCCCGGGTCCTTGTAGAGCTCAGCATTATTGA  ATGAAATCTACTGGTATCGTACGTTATGTATTCTCCTTTCTAAGCTTCT  ACGTACGATACCAGTAGATTTCATTTTCTGTTGGGCCATTGCATTGCCA  CATTAGGCGGGCTGCCCCGGGTTATTCGGTTTCTTCGCTGTCCTGT  ATGGCTTTACTACAAAAAACACGATATGTATTCTCCTTTCTAAGCTTCTG  TCGTGTTTTTTGTAGTAAAGCCATTTCTGTTGGGCCATTGCATTGCCAC |
| Q-rpsU-F | GTCGTTAGAAAAAACGAATCGCTTG |
| Q-rpsU-R | TTGCGTTTTCTAGCAGCTTCTGACT |
| Q-ituD-F | AGTCTTCCGTCGACACTTTC |
| Q-ituD-R  Q-ituA-F  Q-ituA-R  Q-ituB-F  Q-ituB-R  Q-ituC-F  Q-ituC-R | GGTTAAACCGCGCTTTCTTG  GGTCAGTATCGGAGAAGATG  CGTGTTCTGCAGCGTATTTC  CCGAAATGGCAGCCTATTTG  TGGTATTAACGGCTGACTGG  TTGAGTGCGGATGTATCACG  CCTGTAATCACGTCCTCTTC |
| Q-degQ-F  Q-degQ-R  Q-degU-F  Q-degU-R  Q-comA-F  Q-comA-R  Q-sfp-F  Q-sfp-R  Q-yczE-F  Q-yczE-R  Q-glnR-F  Q-glnR-R  Q-abrB-F  Q-abrB-R  Q-codY-F  Q-codY-R  PHT-F  PHT-R | GTGGAAAAGAAATTAGAAG  ATATGAGAATTTATCGAGC  GGCTACTAAGCAGCTTGTTG  TATCCGCGTCCATCTCTTTC  ATCACGTGCTTAACGGACAG  TGTTAACCGATCCTGCTCAC  AGTACGGAAAGCCGTACATC  TCCGGCCTGTTTGATAAAGC  GCTTTAACCTGCCTGTTCAC  GATCAGCAGCAATCCGAAAG  TTTCCAGCGAGAAGCGAAG  TTCGGCAGATGCCTTATCC  TGATGAACTTGGACGCGTAG  CACCAGTTACTTGGCAAGTC  GTCTGGGCACATTGATTCTG  GCTTTGCTTCGTGCTTCTTC  GATGACCTCGTTTCCACCGGAATTA  TTCAGTTGCAGACAAAGATCTCCAT |
| pCB-JC1  pCB-JC2 | CGTTTCTACAAACTCGAG  ACACATTCATGAATTTTCCTCC |

Note: the restriction enzyme cleavage sites are underlined.

**Table S2.** Coded and actual levels of factors used in the experimental design

| Factors | Coded level | | | |
| --- | --- | --- | --- | --- |
|  | Symbol | -1 | 0 | 1 |
| Inulin（g/L） | A | 1 | 7 | 13 |
| L-sodium glutamate（g/L） | B | 5 | 15 | 25 |
| MgSO_4_（g/L） | C | 0.1 | 0.5 | 0.9 |

**Sequence of the six candidate promoters**

**A2cup:**

TATTGCCGATGATAAGCTGTCAACATGAGAATTCCCTTGTAAAACTTTGTCGAACTTTTTATAGAAAAGTGTTGAAAATTGTCGAACAGGGTGATATAATAAAAGAG

**BJ27up**:

TCCGCTCACAATTCCACACATTATACGAGCCGATGATTAGGGGATCTTGTTGTTTTTTTCGTTTTCTTGTATAGTAGAAAGGTAAGAGAGGAATGTACAC

**C2up**:

TGAGAATTCCTAACAACTAAATCACGACTATATACCTATACTATTTATTATCATCAATTTGTCGAAAAGGGTAGACAAACTATCGTTTAACATGTTATACTATAATAG

**P*_amyA_***:

TCTCAGCGGAAAAAGAATCATCATTGCTGGCGGGGGCAATGTTGCATTAAGAAGGCTGAAAACGGTGCTTCCGGAAGGCGCTGATATTACCGTGATCAGTCCTGAGGCCCTGCCTGAAATTAAAAAGCTGGCGGATGAAGGACGCATCCGCTGGATTCCCCGGAGAATTGAAATGAAAGATCTCAAGCCCGCTTTTTTCATTATTGCCGCGACAAATGACCGAGGCGTGAATCAGGAGATAGCCGCAAACGCTTCTGAAACGCAGCTGGTCAACTGTGTAAGCAAGGCTGAACAAGGCAGCGTATATATGCCGAAGATCATCCGCAAAGGGCGCATTCAAGTATCAGTATCAACAAGCGGGGCAAGCCCCGCACATACGAAAAGACTGGCTGAAAACATTGAGCCTTTGATGACTGATGATTTGGCTGAAGAAGTGGATCGATTGTTTGAGAAAAGAAGAAGACCATAAAAATACCTTGTCTGTCATCAGACAGGGTATTTTTTATGCTGTCCAGACTGTCCGCTGTGTAAAAAATAGGAATAAAGGGGGGTTGTTATTATTTTACTGATATGTAAAATATAATTTGTATAAGAAAATGAGAGGGAGAGAAAA

**P_43_**:

AGCTTCGTGCATGCAGGCCGGGGCATATGGGAAACAGCGCGGACGCAGCGGAATTTCCAATTTCATGCCGCAGCCGCCTGCGCTGTTCTCATTTGCGGCTTCCTTGTAGAGCTCAGCATTATTGAGTGGATGATTATATTCCTTTTGATAGGTGGTATGTTTTCGCTTGAACTTTTAAATACAGCCATTGAACATACGGTTGATTTAATAACTGACAAACATCACCCTCTTGCTAAAGCGGCCAAGGACGCTGCCGCCGGGGCTGTTTGCGTTTTTGCCGTGATTTCGTGTATCATTGGTTTACTTATTTTTTTGCCAAAGCTGTAATGGCTGAAAATTCTTACATTTATTTTACATTTTTAGAAATGGGCGTGAAAAAAAGCGCGCGATTATGTAAAATATAAAGTGATAGCGGTACCATTATAGGTAAGAGAGGAATGTACAC

**P*_bca_***:

CCAGCGGACGGAGAGTCCCGAATCGTATGCGCAGTGCGGAAAATGTTGATATAACGGGCTATGAAAAGATGGATTATGATTGCATCTTTTTCTTCCGGAACGCAAATAGTCCATTTAGTCCAAACGAATATCCGTAGGAAAATGTAAACGCATTCATTTTTCTTTAAAAAAATTAAAGTAAGTTCGAAGTCCTGCCTATTCCCAAAAAAAGCAATTCGATAATAGGAAGAATAGCGCCGCTAATAGAGAAGTTTGGCTTAGTGCAGGCGAGGAGATTATGTTACATAATGCCGATTGAGAATTCATAGTGAAGCTATATACTGATGAATGAATTTATCAATACAGAAGGAGATGTCAAAAATCA
